# Institutes of Surgery: Arranged in the Order of the Lectures Delivered in the University of Edinburgh

**Published:** 1838-07

**Authors:** 


### Art. XII.

*Institutes of Surgery: arranged in the Order of the Lectures delivered in the University of Edinburgh.* By Sir Charles Bell, k.g.h. f.r.s. l. & e., &c.—*Edinburgh,* 1838. Two Vols. 8vo. pp. 353, 380.

Placed at the head of a large class of surgical students; enjoying a high reputation; with a mind matured by the active practice of his profession during a period of nearly forty years; it appears natural that the author of the work before us should desire to give his pupils the benefit of his experience in a digested form: and they had a right to expect that the book in which that experience was recorded should contain at least a fair summary of what it was essential they should know of the theory and practice of surgery. It was, we apprehend, incumbent upon him so to frame his guide-book that the students, who attended his class, might fairly lean upon it for support in fitting themselves for the active practice of their profession;—that, though only a summary of what was known, it should be a fair and sufficient summary; and that, in those cases where it left much to be sought for elsewhere, it should have carefully indicated the best sources where it might be found. It is our business to enquire how far and how well these objects have been attained.

The work is doubtless an outline of the lectures which the author has delivered, and many of the blanks which exist in the "Institutes" may have been filled up in the communications made to the student; but the existence of those blanks, and they are numerous, is a serious drawback on its general usefulness, and must materially lessen its value to surgical students at large. There is also a painful want of unity or plan in the work. A large portion of the subjects are merely shadowed out, whilst a considerable number are tiresome from their minuteness of detail: thus, to Mortification, including its nature and treatment, two short pages are allotted; to Phlebitis, one; to Caries, that most important disease to the surgeon, twelve lines are devoted; while Scarifying the Gums boasts of two pages, and Pivoting Teeth of one. Again, at the end of the second volume we find an "Appendix" of 121 pages of small type, containing more than a third of the matter in the whole work; this space is occupied with clinical lectures (delivered at the Middlesex Hospital, and long since published in the Medical Gazette,) and a large number of cases, which, if they appear at all, should have been placed in the chapters which treat of the diseases to which they refer.

We are most sincerely grieved to notice these, and, we regret to say, yet greater blemishes that are to be found in this work; because the unanimous voice of the present age and of posterity must hail Sir Charles Bell as one of the lights of medical science. His labours and great discoveries in Physiology will render his name immortal, as they now render it revered in every country of Europe. Why, then, does he force upon our attention, in many parts of these volumes, a narrowness of mind and illiberality of feeling so unworthy of his intellect and his fame? The following, is an example. "In noticing a few authorities," he says, "I have preferred *English* authors, principally because they stand distinguished by truth of narration." (*Introductory Chapter*, p. 23.) We deprecate, in the strongest terms, this insinuation as to the good faith of foreigners. No matter what their character or country, they are involved in one sweeping condemnation as persons whose statements are unworthy of credit. What right have we to arrogate to ourselves exclusive purity? Is truth never violated in our own land? No doubt there are in all countries persons whose veracity may be impeached; in our own, we regret to admit, that many such may be found; but would this circumstance palliate the injustice of that person who should stigmatize us as a nation of liars?

In the notice which we propose to take of this work, we shall so far follow the sketchy and desultory manner of the author as to put down, without much regard to connexion or continuity of narration, such remarks as occur to us in its perusal. We shall have occasion to condemn as well as to commend: and here, as in all other cases, we shall be influenced more by what is due to science and our readers than by any personal regard we have for the author.

At page 7 is a passage which astonished us; the author says "it will not be imagined that, after the life the author has led, he can desire to throw discredit on morbid anatomy; but, by pottering in the dead house we shall not discover the sources of disease." It certainly is difficult to imagine that any man holding the office of professor of surgery, and charged with the direction of the studies of a large number of young men, should have deliberately penned such a sentence. What should be its natural effect, but to impress the student with an opinion that it was unnecessary to devote much time to the prosecution of this branch of medical knowledge? What can be more contemptuous than this expression, coming, too, from a person who has profited so largely from morbid anatomy?

The third chapter is on *Adhesion.* In speaking of the means of procuring adhesion, the following judicious remarks occur. "All that regards dressing should be studied in the hospital. But from the bad taste (to give it the mildest term) which prevails, and which tempts surgeons to contend against time, and therefore to have the patient soon off the table, the dressings are huddled on or deferred. Surgeons of public institutions owe therefore a public duty,—to serve the poor, but also to be an example to their younger brethren, that right methods may be taught. The dressing of a patient after an operation is very often the most important part of the office of a surgeon, and anything like hurry the most dangerous lesson." (Vol. I. p. 18.)

On the subject of *Whitlow,* we differ from the author in his opinion that it originates in an inflammation of the vascular body which secretes the nail: in the majority of cases it may be so, but in a considerable number of instances it is entirely unconnected with this organ. With respect to the treatment of this complaint, he observes:

"When the matter is deep, says an old surgeon, there is no alternative; place the patient's hand on a mass of tow, hold him firm, and with the scalpel cut to the bone! All I have to say is, that it may be done effectually, and with less pain, by passing inward a fine sharp bistoury, and cutting outward. Cut away the nail; touch the ulcer, and especially the very sensible fungus, with the blue-stone; and, according with the old advice, dress with the balsam copaibæ and tinctura thebaica." (p. 27.)

In treating *Chronic Abscess,* the following few plain and useful suggestions on the mode of opening them occur:

"When you puncture an abscess, lay the flat head of your probe on the side of the lancet, and hold them firm. When you have penetrated to the matter, by a motion of your thumb introduce the probe further, and withdraw the lancet; or you may use the directory in the same manner; the groove of that instrument serving the purpose of a canula. A grooved needle is employed for exploring a tumour in which the surgeon is uncertain whether there be matter contained or not. The use of the probe or directory is to permit the matter to flow, and to prevent the necessity of much poking in the wound, which makes the union of the puncture uncertain." (p. 29.)

In the treatment of abscesses, to prevent their falling into obstinate sinuses, the following reasons for the necessity of rest are given.

"I have found an abscess under the latissimus dorsi and under the trapezius, kept open by permitting the motion of the arm. Mr. J. Bell was wont to narrate a case of fistula under the pectoral muscle, which had not yielded to injections, setons, compresses, &c. cured by the necessary bandaging on the patient's breaking his arm. When there are abscesses in the temple, it is necessary to keep the temporal muscle at rest by binding up the jaw. Sinuses after bubo in the groin are in like manner kept up by the motion of the part, and cured by strict rest, compress, and bandage." (p. 31.)

We have already remarked on the insufficient space given to *Mortification.* The chapter commences with the following observation:

"Looking, as we do here, directly to practice, and eschewing theoretical discussion, we say, mortification is one of the terminations of inflammation; but it is essential to observe that when phlegmon terminates in the death of the part, there is an essential omission in authors; it is the effusion and infiltration into the cellular membrane which checks and hinders the action of vessels." (p. 38.)

In Dr. Carswell's "Illustrations of Pathological Anatomy," Fasciculus vii., published in 1835, (and similar statements are made by several other authors,) is the following observation on the subject of mortification: "these appearances would seem to prove that the rapidly destructive effects of this form of inflammation depend, in great measure, on the mechanical influence exercised by the effused fluids on the capillary circulation. These fluids must compress the neighbouring veins to a degree that will prevent the return of the blood poured into the capillaries." So much for the "essential omission in authors."

In the following page it is stated, that the cause of *gangrena senilis* "is ossification of the arteries of the leg;" that "it commences in a small black spot on the side of the little toe." Here, again, we refer to Dr. Carswell, and we find "that, in every case of gangrena senilis which I have examined after death, the arteries of the limb were obliterated to such an extent as to interrupt the circulation of the blood. The obstructing cause consisted, in five or six cases, of a fibrous tissue formed either in the walls or cavities of the arteries, and which had converted these vessels into nearly solid cords of ligamentous consistence." There is no doubt that, in old persons, who are the subjects of this disease, ossification of arteries would be found, but not often, in the leg itself. MM. Dupuytren and Londe incline to the opinion that it is always a consequence of arteritis; but this is not proved. With respect to the point at which the spot first appears, it is a question of fact to be decided by the observation of every medical man. In Sir C. Bell's experience it may have been the little toe; in the experience of other persons, this preference for the little toe has not been always observed.

In the next section of the same chapter, *Anthrax* is treated of very satisfactorily: were the other portions of the work as well done, our present task would be one of unmixed pleasure.

Chapter vii., *of Wounds*, gives also a good practical consideration of the subject. There is one point in it upon which we shall reserve our opinion until we come to the chapter on Amputation—the use of needles and ligatures.

Next comes the important subject of *Hemorrhage*, and this is treated with much ability: in fact, this is one of the author's best chapters, and is evidently written *con amore*. At page 52 he states, that "many years ago he showed that, if an artery was bleeding during an operation, and you took your forceps and pinched it, it would soon cease bleeding. The intelligent reader will see how this touches on the subject of the *torsion* of arteries, which is nothing more than a mode of injuring the coats." Does the author mean, by this passage, to insinuate that the idea of

*torsion* came from him? Long before Sir Charles Bell pinched arteries with a forceps, it had been done by others: but this *pinching* will not restrain hemorrhage, except from very small arteries, and not at all in those of a fifth or sixth rate caliber; and, in principle, it is very unlike that upon which *torsion* is dependent. In the latter, the vessel is twisted upon itself so completely that a fair section of the internal coat is made, and a physical obstacle to the passage of the blood is formed, even in the axillary artery; and this so powerful, that we cannot succeed in establishing the canal, even with the assistance of the propelling power of a large syringe. It is probable that the ancient surgeon who attended to the following advice of Galen often *injured* the coats of the artery while *pinching* them: "Si vas undè profluitur alte sit demissum, certius ipsius tum positum intelligat, tum etiam magnitudinem; præterea vena ne sit, an arteriola. Post hæc injecto unuo attollat." (*De Loc. Aff.* lib. i. cap. i.) In Peyrilhe, also, (*Histoire de la Chir.*, tome ii. p. 638,) we find the following sentence: "Si le vaisseau qui fournit le sang est profond, le chirurgien tâche d'en reconnaître la position et la grandeur, de distinguer s'il est veineux ou artériel; il le soulève ensuite avec un crochet, et le tord un peu." Even for a century back (perhaps for many centuries) it has been the custom of sow-gelders, in the practice of their art, to restrain hemorrhage by twisting the vessel five or six times. Most persons are aware that M. Maunoir, of Geneva, published a series of experiments on the subject, in the year 1820. At page 53, Sir C. Bell says "a man of genius values what he has discovered, and respects the thing in others. Dr. Jones was wrought upon to be unjust: his external and internal coagulum were matters better explained by Mr. John Bell, who also showed the danger of trusting to this state of the vessel." It does not appear to us that it was necessary for Dr. Jones to have referred to Mr. John Bell. Our own opinion is, that Dr. Jones's observations were much the better of the two: besides, as far as discovery is concerned, it does not rest with either of these gentlemen. In 1733, J. L. Petit communicated to the Académie des Sciences the results of certain experiments which he had made upon the obliteration of arteries. According to him, there is formed, at the divided extremities of the vessel, two clots; one of which surrounds and presses upon the extremity of it, the other penetrates as a central plug. In 1736, Morand modified this theory, by stating that the artery suffered a retraction, accompanied by a circular femoral contraction, which served to "imprison" and fix the clot. Variously modified and expressed, this explanation has descended to the present day: to whom, therefore belongs priority? If Dr. Jones referred it to any one, it clearly should not be to Mr. J. Bell.

The following observations are accurate and judicious:

"The ligature puts a mechanical stop to the hæmorrhage; it also stimulates to the reaction of the living vessels, and the adhesion and final obliteration. In this double office it should be considered, to arrive at just principles. At the time when writers on surgery ran away with the idea that the action of a ligature was to bring the inner coats of an artery into the state of an 'incised wound,' I foresaw what follies would be committed; and at that time I had a large class of intelligent students, I made the experiment to show that it was the presence of the ligature as a foreign body that excited the coats of the artery. I placed a circular ligature around the artery, letting it lie in contact with the proper coat of the artery, without drawing at all, and showed them the artery stopped by a clot." (p. 56.)

"Mr. Lawrence, in the Medico-Chirurgical Transactions, vol. vi. page 156, treats of some important subjects, but the chief part of the communication is on the ligature of arteries. He is the advocate for small, hard, single thread ligatures, which he cuts off short by the knot. This was a practice of John Bell's, and which I did not approve of then, nor do I now. The object is to procure immediate adhesion, which, it is alleged, is prevented by the common manner of retaining the ends of ligatures. I have seen patients, under the hands of good surgeons, shrieking and complaining of their toes, when ligatures were put on the arteries on the face of the stump. In matter of fact, the ligature often includes the nerve. Conceive, then, the misfortune of having a fine, hard thread firmly tied on a nerve, and the ends cut off; conceive the distress, the pain, the tic, thence arising! and you will not follow this fashion, for it is but a fashion. Long after the stump should have been well, ulcerations have been occasioned by the ligatures being cut short, and the noose left." (p. 58.)

"The conviction that my brother's conversation and practice left upon me was, that the artery should be firmly tied with a moderate-sized ligature; that the surrounding parts should be disturbed in the least possible degree; and care taken that, whilst the thread was within the sheath, and in contact with the proper coats of the artery, the vasa vasorum were to be preserved entire." (p. 62.)

Chapter ix., which treats of *Gun-shot Wounds*, deserves unqualified commendation. In speaking of the occasional difficulty of discovering a musket-ball, he says,

"The nerves will sometimes indicate the course or position of a ball. I might have said, in the last case, that, by the numbness in the course of the radial nerve, on pressing against the ball, I had informed myself that, although it lay apparently superficially, the nerve ran over it. So, when a ball has taken its course through the pelvis, or across the shoulder, the defect of feeling in the extremity, being studied anatomically, will inform you of its course; that it has cut or is pressing on a certain trunk of nerve." (p. 78.)

His statement that he "never saw a living man with his thigh carried off," must not convey an idea that the case does not occur; for there are many such on record, and on indisputable authority, in which the patient recovered.

The chapter on *Tetanus* (x.) is as meager a sketch as could well be conceived. His description is as follows:

"One of the most alarming consequences of wounds is tetanus. This consists of violent and permanent contractions of the voluntary muscles, and consequent tension and rigidity." . . . "As the cases have presented themselves to me, my attention has not been called to premonitory symptoms, though such might have been observed: dejection, and an anxious countenance, mark the commencement of the attack; the patient feels a stiffness and rigidity of the muscles of the jaw; the tongue is affected." (How?) "This rigidity extends to the abdominal muscles, with a pain striking inwards, as if the diaphragm were affected. The spasms become more universal, the more powerful muscles constraining the less." (pp. 84-5.)

The treatment is still more vague, and the references are to an essay by Dr. Maclagan and Sir George Ballingall; no notice being taken of Trnka, Larrey, Dupuytren, or the recent clever compilation of Mr. Curling.

From the chapter on *Ulcers*, which is a very good one, we extract the following graphic description:

"An ulcer is attended with an absorption of the adipose membrane beneath and around it; its edge is elevated by deposit of coagulable lymph, and these together make the sore appear deeper than it is actually. Its surface is covered with a film of coagulable lymph, and in this the changes are wrought which we have to notice: in this lymph the granulations are formed. The granulations are those small, red, convex bodies which appear on the surface of the ulcer: they secrete, and cover themselves with, pus." [Or rather, we should say, the pyogenic membrane which covers them secretes the pus.] "You may see the granulations forming on the inflamed surface of a bone. You perceive, on one day, a matter on the surface like pale jelly, which you might wipe off: on the morrow it is fixed, and, when you touch it, it bleeds." . . . "It is of importance to notice the *welt* around the ulcer; for, while it continues, the ulcer will not heal. It has been described as a circumvallation, which the ulcer throws up for defence: the idea is absurd, but it leads to a useful inference that you must cause the absorption of this hard circle, or it will be in vain to attempt healing the sore." (p. 87.)

In Chapter xii., *on Diseases of Bone,* Sir C. Bell says (p. 97), "I do not object to the term *periostitis:* but I believe it to be a mistake to describe the periosteum as the seat of a disease distinct from the affection of the bone itself." We apprehend that the mistake rests with the author. In many cases of periostitis, the bone is not affected, in those cases more especially where the disease has been induced by the vicinity of chronic ulcerations. We have lately known three cases in which the bone was untouched: in one, the periostitis was developed spontaneously on the inner border of the tibia, without any change of colour in the skin; the second and third cases were in the same bone, lower down: there was no purulent matter present in either, and the membrane adhered to the bone in the ordinary way. We are aware that the opinion of our author has been maintained by others. Hunter believed that the bone is primitively affected. We are not commonly called upon to examine such cases until the bone has partaken of the disease; but, if we attend to the seat of pain, and to the organic changes which are brought about in the periosteum, we cannot refuse to acknowledge that the disease first attacks the periosteum.

That most important disease, *Caries,* is disposed of by the following paragraph!

"Much has been written to little purpose on caries; because they do not define what is meant. A surgeon puts his probe into an ulcer, and he finds that he can force it into the bone, and he says 'The bone is carious.' What is this? A bone softened by inflammation, where the earth has been in part absorbed. Or, again, in probing, he feels the bone rough: 'The bone is carious.' In this latter case the bone is dead; deprived, consequently, of its coverings and periosteum; it is exfoliating. Or suppose that there is an ulcer of the leg which extends to the bone: 'the bone is carious.' Thus is caries anything or nothing: the bone may participate in any disease modified by the peculiarity of its composition, in possessing the hardening material, earth or bone." (p. 98.)

Is this such a summary as we have a right to expect in a work written by a professor of surgery, as a guide or code of instruction for students?

Again, in *Necrosis,* (p. 100,) the subject is just as loosely treated. Sir C. Bell says, "There is no reasoning on the subject of necrosis, unless we go to the elements of anatomy, in which very few are properly initiated." But, surely, because the author entertains this opinion, he is not therefore to be exonerated from entering upon the subject; nor the student to be sent to practise his profession in perfect ignorance of this frequently-occurring and formidable disease?

Again, in p. 102, all the description given of *Mollities Ossium* is the following: "In that case there would be pains universally in the bones, with earthy deposit from the urine. The disease proceeds, I am afraid, unchecked by remedies. The disease is very slow in its progress, which therefore makes the observation of the effect of remedies difficult." Now, what idea of the nature of this disease can the student derive from such a description?

*Fragility of Bone* does, we apprehend, exist under other circumstances than "from want of use and old age;" but we have no room here to enter upon that subject. The cancerous and scorbutic diatheses appear to predispose to it. The experiments and observations of Troja show that it does not depend upon a superabundance of the calcareous phosphate or a diminution of gelatine.

Chapters xiii. and xiv. relate to *Fractures;* and, with considerable defects, contain much valuable practical matter; but, as we shall shortly notice the whole subject, in a separate article, we pass it over for the present.

The portion of the work which treats of *Diseases and Injuries of the Spine* is entitled to much praise; but even here, good as we deem it, we have to regret that the author seems to be unaware of everything done by younger men than himself. We could have wished that he had expressed an opinion upon the mode of treating diseases of the spine by laying the patient on his belly upon an inclined plane. By this plan certain important objects are attained: the patient being kept there night and day, you secure quiet; the feet and the arms hanging down, exert some straightening influence; no pressure is made upon the curvature; and applications of any kind may be made with great facility. We have recently seen this plan in operation, the children being very cheerful and satisfied, feeling no restraint; and the functions of the abdominal viscera much improved by the pressure made upon them.

Among the cases of fractured spine which he gives, it is singular that Sir C. B. did not allude to that communicated by Mr. B. Phillips to the Medico-Chirurgical Society, and published in the Society's Transactions. In this wonderful case, the man fell from a hayrick upon the back of his head; he was stunned by the fall, but soon recovered; he lived eleven months, and the only discomfort which succeeded the injury was an inability to rotate the head. After death, it was found that the violence had fractured the atlas transversely; the anterior part of the ring was driven down between the pharynx and the spinal column, until it came on the same plane with the axis, to which it became attached by perfect bony union; the transverse ligament which attaches the processus dentatus was strong enough to carry that process with it: and yet all this violence occurred without occasioning any important or distressing symptom!

Sir C. Bell is incorrect in stating that authors have considered dislocation of the spine in no other light than as producing pressure on the spinal marrow. It is true they set it forward as the most prominent evil, and it is so; for pus is not always formed as a consequence of such injuries; for instance, it was not even in Mr. Phillips's case: neither does inflammation of the spinal marrow always occur. He will also admit, and should have done so already, (as it appears that the singular vertebræ in Mr. Phillips's case, as well as its history, is in his possession,)

that, "when the fracture is above the principal origin of the phrenic nerve, the act of respiration is [not] always stopt; and that death from suffocation does [not] always suddenly follow." We are free to admit that the cases in which an attempt has been made to elevate a depressed portion of the spine have not been very successful. We know of only four cases; and, of these, one was performed successfully, as we are informed, only a few months ago, by a surgeon of the name of Edwards, living at Caerphilly, in South Wales. There were present the usual symptoms of compression,—paralysis of the organs of locomotion, the rectum, and the bladder: the situation, as far as the operation was concerned, was unfavorable,—the lumbar region; the posterior arch of the bone was raised, the symptoms of compression relieved, and the patient did well. As far as other circumstances were concerned, the situation was indeed favorable; for it may have been at a point where the medulla spinalis no longer retains its cord-like form, and where pressure is less suddenly fatal in its effects. How long Cline's case survived the operation, we do not know. Tyrrel's died of peritonitis, at the end of three weeks. Barton's patient lived only four days; but during that time there was a momentary return of sensibility. The operation therefore is not, as Sir Charles states, "inevitably fatal." We are unable to comprehend the grounds upon which the author assumes "that the membranes of the spinal marrow are the most susceptible of inflammation and suppuration of the whole frame." (p. 157.) In our ignorance, we should have said, without hesitation, that pleuritis was more commonly seen than spinal arachnitis.

*Injuries of the Head* occupy the sixteenth chapter; and with it neither ourselves nor the student have cause to be dissatisfied. On the subject of compression, to which the author has given much attention, there are some ingenious remarks, which, although we do not entirely adopt, we may refer our readers to. The author states "that there is a hiatus in all writers on wounds of the head; they take cognizance of no other cause of torpor than compression." He should have stated that he supposed this must be so, but that he had not ascertained that the statement was correct. It happens that most writers on the subject distinctly point out that torpor may exist without compression : for instance, it may accompany the incubation of inflammation which has been excited by simple concussion.

The section on *Hernia Cerebri* is most unsatisfactory: why not either have referred to or condensed the able memoir on the subject by Louis?

In chapter xviii., where he speaks of *Injuries to the Hip-joint,* he should have stated that the changes produced in the head and neck of the femur by "inflammation of the hip-joint" are also produced by external violence, by a fall on the great trochanter, and that it is described satisfactorily by Mr. B. Bell, in an essay on Interstitial Absorption. All that is said under the head of *Compound Dislocation of the Knee* is, "This is a case for amputation." In the section on *Dislocated Ankle,* he has omitted two cases which may be found in the fourteenth volume of the Medical Gazette, and which are quite unique. By jumping against a bank in one case, and falling forwards, the dorsum of the foot was forcibly brought towards the tibia, and the astragalus alone was driven backwards, until it got clear of the articulation, and, without breaking the skin, caused the tendo-Achilles to describe an angle of sixty-five degrees.

In *Dislocations of the Humerus* at the shoulder, the author very properly alludes to simple modes of reduction. The following is one in which a person, unassisted, may almost always succeed in reducing it:—Let the patient sit sideways in a chair, and let the injured arm be brought over the back of the chair, which has been previously padded for the axilla: take a round towel; make a noose by passing the end of the towel through, and fix it upon the arm just above the elbow: you then place yourself at the back of the chair; of the depending part of the towel you make a stirrup, into which you put your foot, and make as much pressure as may be necessary: you have thus both hands free to manipulate the joint.

In *Dislocation of the Thumb*, Sir C. B. seems to be desirous of impressing the student with the conviction that he had better not use violence in reducing it, because the second joint "has been torn off in reducing the first." We agree with him that such violence is improper; but it is still very desirable that the dislocation should be reduced, and he should have been prepared with some substitute. Mr. Liston states that he had succeeded by cutting the lateral ligament.

In speaking of *Club-foot*, he says, "on dissection, I have found the defect less than could have been imagined." Our own impression is different: it is true that a considerable deformity may exist without occasioning anything more than a change of direction, in the phalanges of the toes, the metatarsal, and even the three cuneiform bones; without, in fact, any change of configuration. But, when we come to the scaphoid, the cuboid, the astragalus, and the calcaneum, the case is different. Scarpa held that the scaphoid was most deformed, but this opinion is certainly incorrect: it suffers little or no change in configuration, but much in direction; the greatest change which happens in the scaphoid is a loss of bulk, or atrophy, produced probably by the pressure consequent upon its unnatural position; the cuboid is similarly affected. The astragalus suffers the greatest deformity: there is here not only change of direction, but also of conformation; it is usually diminished in volume; it is always deformed. The calcareum follows the astragalus in its displacement, only its external border rests on the ground. The plantar fascia, the ligaments, especially the internal lateral, and the muscles, also suffer considerable changes. How this state is produced, it is not easy to determine. "Mechanical means" will not always cure this deformity; and it is singular that the author has not pointed out another mode of treatment, at this moment much employed, and certainly with great success in numerous cases. This operation consists in the section of one or other of the tendons of the muscles of the leg, which allows of a ready reduction to the natural state. A large number of cases of this kind are now on record, many of them of very old standing; and, although some of them may have been curable by well-directed mechanical means, yet there are others which would not have been so cured; and in all cases the time occupied is much less. It would be desirable, but difficult, to determine the circumstances under which we may cure club-foot without section of the tendon. By the hand alone, in infants, we can change the direction of the parts, and the section is unnecessary. And, in this case, they may be readily enough maintained in their new position by plaster of Paris.[*] In after-life, we may also dispense with the section wherever the muscles will give way upon the force which may be applied by the hand; but, when this cannot be effected, the section of the tendon will enable the practitioner very soon to restore the foot to its natural direction. Another reason why this method should be employed is, that the operation is not nearly so painful as the extension procured by brute force, and it seems to be unattended by danger.

The second division of the volume commences with the ***Diseases of the Natural Passages.*** In speaking of ***Ranula***, he says, "I have taken the patients to old and experienced practitioners, to ask what they called the complaint? They have answered, Ranula! I have then passed the Anel's probe into the sublingual duct, showing it to be quite pervious: therefore, I do not give the definition 'a tumour under the tongue, from an obstruction of the duct.'" (p. 218.) No one knows better than Sir C. Bell that a calculus may pass along the urethra, that it may be detained at a certain point, that it may obstruct the passage, that a pouch may be formed for it, that it may glide into it, and that a bougie may then be easily passed along the urethra: why may not a similar circumstance occur to the sublingual duct,—for cretaceous particles are often found in ranula? But we look at it under another aspect: we apprehend that ranula is usually understood to be a tumour which results from an accumulation of saliva in the excretory ducts of the *submaxillary* glands; rarely in those of the sublingual. However, we are free to confess that, whether these tumours be serous cysts in contact with the parietes of these ducts, or in direct communication with them, is a fact yet undetermined. The disease is an obstinate one, and difficulties are often experienced in curing it. Besides the methods of treatment alluded to by our author, there is one which was invented and successfully employed by M. Dupuytren. The instrument he used was a little hollow cylinder, terminating at each end by expansions something like two buttons joined together by a smaller central portion; a small snip was made in the tumour, so as to admit with difficulty one end of this instrument, which was introduced and left there. It was afterwards modified by making it solid, it being found that the saliva escaped around its circumference.

The section on ***Stricture of the Œsophagus*** is important.

> "I have been in the use of passing the bougie at regular intervals, so as to preserve the passage free. There is another method, effectual and safe; by using a kind of probang, with a conical piece of sponge. This, dipped in a weak solution of nitrate of silver, and passed repeatedly through the stricture, dilates it, clears away the mucus, and removes the disposition to spasm. The patient swallows with comfort for a long time after this process. But to the question we must come at last, what is to be done when a wretched creature is actually starving, in consequence of stricture in the œsophagus? I look in vain for authority and advice. I have attempted, by incision on the side of the neck, to divide the stricture and use a tube, but failed." (p. 243.)

With respect to dilatation by bougies, it is true a certain quantity of relief is thus afforded, but we fear this is all. There are some cases on record in which a cure has been accomplished. Paletta mentions one case, Home another, Earle a third. Mr. Fletcher has used a threebranched dilator, but it is an unscientific instrument. Costallat's plan has also been recommended. Four times, in a solid form, nitrate of silver was applied "successfully" by Home; three times unsuccessfully; three times also by Andrews, and in one alone did it succeed. We cannot feel satisfied that these were genuine cases of stricture. If the stricture be a consequence of chronic inflammation, of induration of the mucous or submucous tissue, cauterization may be attempted; but how can we be sure that it is not cancerous, fungous, &c.? and, in that case, what would be the use of such an application?

On the subject of ***Phthisis Laryngea*** he says, "I long since, in hospital reports, recommended the solution of nitrate of silver to be squeezed into the glottis, by means of a piece of sponge attached to a catheter wire. The late Mr. Vance adopted the practice." (p. 250.) This passage would seem to convey the idea that Sir Charles was the originator of the practice. This may have been the case; but it is well known that Mr. Vance employed it long before the publication of "Hospital Reports." But, for a fuller consideration of this subject, we refer the reader to the article in our last Number.

At the commencement of the section on ***Diseases of the Prostate Gland***, the following judicious advice is given to the student:

"The student of surgery has his most important lesson to get from the dead body. He should examine the outside and neck of the bladder, the place of the entrance of the ureter, the relation of the vesiculæ seminales and prostate gland, the distance of the prostate from the anus. He may consider how these parts should feel with his finger in ano in the living body. He should open the bladder, and pass his probe along the mucous membrane, thinking of the possibility of natural obstruction to instruments, and the place of diseased obstruction." (p. 285.)

This section is, however, on the whole, unsatisfactory and incomplete.

The section on *Irritable Bladder* contains some good practical remarks. In the case of children who pass water when asleep, Sir C. B. repeats a recommendation made by him long since.

"The position of the child in bed should be attended to. When we lie on the back, the urine gravitates to that sensible part of the bladder on which the action of the muscles depend. The sensible spot on the interior of the bladder, just below and posterior to the opening into the urethra, commands the muscles at the neck of the bladder as infallibly as the sensibility of the glottis controls the muscles of respiration. When that spot is irritated, you are forced to make water. If you order that the nurse should lay the child on its cheek, inclining it to lie on the stomach or the side, there will be no call to urine, no wetting of the bed. On giving this rule to older patients, they have found it infallible; so much so, that they would start up the moment they were sensible that they had turned on their back, knowing the consequence." (p. 293.)

***Stricture of the Urethra*** is noticed somewhat in detail, as its importance deserves. "The permanent cure of stricture, (says Sir C. B.) is to be accomplished by dilatation." If, from this sentence, the student should infer that stricture is, or may be, permanently removed by this means, he would be in error. A certain, but not a large, number of obstructions in the urethra may be removed by the judicious employment of dilating bodies; but, if a stricture have existed long, and if it be dependent upon a mass of induration occupying the submucous tissue, the only *cure* which will ordinarily be obtained by such means is a *temporary* dilatation of the canal. We say temporary, because the tendency to contraction remains, and will be soon developed, unless the use of the dilating instrument from time to time (that is, with intervals of perhaps a fortnight,) be persisted in. The statement, therefore, "that the membrane has no disposition to contract," is, we believe, incorrect. We do not think that the presence of the dilating body for ten minutes is enough, unless the urethra be irritable: many men express no inconvenience at the end of half an hour; and, when this is the case, it would be better to retain it so long. We agree with the author that extensive applications of caustic are rarely necessary, and that they must be followed by a tendency to contract, in consequence of the generation of fibrous tissue; but we do think that there are modes of applying it very preferable to the armed bougie. Allusion should have been made to the modes of using it recommended by Ducamp, Lallemand, and Phillips. We apprehend that the stimulus given by a slight application of caustic,—merely brushing over, in fact,—should be soon followed by the dilating body; as, by this combination, the absorbents are more efficiently excited to the removal of the indurated matter than by simple dilatation.

Sir C. Bell has lived long enough to modify an opinion once expressed, now many years ago, that no stricture could exist at or near the prostatic portion of the urethra: in the present work, it is so put that the truth of his position depends on a definition. We are not, however, disposed to quarrel with any man for abandoning a position which is no longer tenable; we are desirous only that opinions should never be too positively maintained. In the works of M. Lallemand and Mr. Phillips are several cases in which the obstruction existed between seven and nine inches from the orifice. Some of the obstacles no doubt occupied the prostatic portion of the canal; but we are quite aware that we may be met by the statement that the obstruction was caused by Cowper's glands, the sinus pocularis, or, in fact, by anything but by a stricture in or near the prostate. M. Lallemand, however, gives the post-mortem examination of a person who died during treatment for urethral disease. "Within a few lines of the prostate, (he says,) we remarked projections, hard to the touch, about five lines long, and of the thickness of the nail; they appeared to be evidently the debris of the principal stricture."

That "there are men whose hourly business is poking into this passage with bougies, who know nothing of its structure or diseases," is unfortunately too true, but, in every period of civilization, pretenders will exist. It is still more to be regretted that many men who do know its structure are accustomed "to force" strictures, and rupture the membranes. This is a reproach to which our author is not open; we have often admired the great delicacy with which he used instruments in this canal. This tact, however, was not more than is absolutely necessary: very few cases can arise to justify force, or in which gentle and patient treatment will not accomplish all which can be done by force, and without its evil consequences.

The best chapter in the work is unquestionably that *on Amputation.* The principal points are here more carefully considered, the omissions fewer. No student could read it without improvement; no practitioner without satisfaction. As reviewers, however, it is still our duty to consider the propriety of certain of the instructions which are laid down, and to point out the reasons which induce us to withhold our entire concurrence in the positions which are taken.

"An amputation," says Sir C. B., "is well performed when these objects are attained:—1. The skin so cut that it shall cover the muscles. 2. The muscles covering and concealing the end of the bone. 3. The bone sunk deep with all its periosteum about it. But, besides these, there are other very important circumstances which innovators forget—the ligature of the arteries, and the condition of the nerves." (p. 323.)

Why that fling against innovators? it is unnecessary, and not generally applicable; besides, in many situations in the present volume, he prides himself on his innovations; and why should he not? to innovate successfully is great merit.

We make the following extract simply because the subject is very important, and because anecdotes, as illustrations, have often more force than simple directions or suggestions.

"You look around, as you ought in all the principal operations, to see that every thing is prepared. Will you believe that the late Mr. Lynn, of the Westminster Hospital, on putting out his hand for the saw found there was none! and they had to send to the joiner. That, on another occasion, in tapping a woman, the foolish assistant gave him the stilette, and kept twisting the canula between his fingers, which was not discovered until the surgeon had plunged the instrument into the woman's side! Recollect what befel a good man, that on operating for the stone, and having made his incision, there were no forceps—no, nor within twenty miles of the place. From that time the gentleman resigned his profession, and all men pitied him." (p. 324.)

He insists, as he has formerly done, (and a large number of the profession have at last acknowledged the justice of the opinion,) upon the necessity of a particular position of the limb when the bone is sawed through.

"The pupil who has sat holding the leg now rises, and raises the limb so that the thigh-bone is perpendicular. You touch with your knife as the muscles withdraw themselves from the bone, especially on the back part, and at the linea aspera. If you at this juncture take a general view of the parts cut, you ought to see, towards the leg, the skin retracted off the fascia, and the muscles projecting, and about two inches of the bone bare." (p. 326.)

The bone is then sawed through close to the integuments. "When the amputation is thus performed, and when you bring down the stump, the bone has disappeared entirely among the muscles; you see it no more, unless you again raise the stump to the perpendicular. When you bring the integuments over the muscles, they fall together neatly, their edges exactly corresponding, and without the slightest irregularity." (p. 326.)

The author's estimate of the comparative merits of the circular and flap operations is very good, and is singularly contrasted with Mr. Liston's consideration of the subject, who does not seem aware of the existence of any other method of amputation than the flap operation.

"Let us consider the different conditions of the face of the stump. The muscles being cut obliquely, and in some measure drawn out by the knife, the integuments (which has a thin edge) is too short to cover them. You will see this exemplified in the flap operation below the knee, where the parts being cut from within, the gastrocnemius hangs out beyond the skin. The muscles being cut irregularly and obliquely, the arteries are not easily found, and their mouths are of the shape of a pen; whereas, in the other mode of operating, their mouths present directly and are easily found in the flat muscular substance. But all objections are of no consideration compared with the state of the nerves. They give less resistance, are drawn out before the edge of the knife, and hang from the face of the stump. I have seen it necessary to cut off a full inch and a half of the popliteal nerve from the face of the stump. I have been brought to reflect on this subject, because I have never seen so many cases of affection of nerves in the stump as in the last years of my attendance on the Middlesex Hospital, which I attributed to the mode of operating with the flap. I had long made it a particular injunction on my pupils, that when they amputated in the common way, they should on retracting the integuments, be careful to cut short the cutaneous nerves which run upon the exterior of the fascia, and not to leave them with the integuments; and this I did under the conviction, from experience, that those sensible cutaneous nerves being engaged in the cicatrix, gave rise to great distress. You will perceive that, in the flap operation, these cutaneous nerves are left long, projecting to the very edge of the integuments. In whatever way you amputate, these dissections" speaking of those contained in a paper of Mr. Langstaff's in the 16th vol. of Med. Chir. Trans., "as well as the sufferings we witness in patients, evince the necessity of burying the nerves deep in the stump. I have often referred to this subject at clinical lecture; for, unless the surgeon attend to this matter (and they do seem to think very little about it) the patient who has suffered amputation becomes an invalid,—a sort of barometer exhibiting the change of weather. After these observations, you will perceive how I consider the question to hinge as to the mode of operating. A clever operator will make a good stump either way." (pp. 330-331.)

Now, with respect to dressing, which, as is said, is of far more consequence than the mode of operating, Sir C. Bell advocates the old system "much dressing and careful rolling." And no doubt in discreet hands this will do well enough; though we confess, we like the simplicity of that employed by Mr. Liston.

In speaking of *Amputation of the Stump*, he says,

"M. Maunoir, of Geneva, published a memoir in favour of the English mode of operating in amputation. In that essay he contrasts the method of which he is the advocate with that practised by Dupuytren, who, having divided the integuments and muscles together, took the farther means of ensuring a bad result by raising up the stump to the perpendicular; and this he did for a reason somewhat too ingenious,—that the blood might be impeded by the sudden angle of the artery at the groin and the ligature protected." (p. 336.)

We are not aware of the existence of any such reasons in the published lectures of Dupuytren. In justice to that great surgeon, we will give the reasons which influenced him in cutting "through the muscles and integuments together." In speaking of various modes of operating, he says, "of all these modes, some attain only very imperfectly the end proposed; others are difficult to execute; and, by modifications newly adopted, the sufferings of the patients are uselessly prolonged. In dividing at first the skin, in afterwards dissecting it, then in successively cutting the skin, the superficial, the propound muscles, and, lastly, the muscular fibres adherent to the bone; it is evident that the instrument is applied three or four times to the part, and the operation comparatively long and painful." This method, in the hands of Dupuytren, at the Hôtel Dieu, was certainly very successful. By a single incision, he cuts at once perpendicularly upon the bone. Sometimes, however, the incision was directed obliquely after the mode of Alanson; the retraction made by the assistant, and the contraction of the muscular fibres, give instantly to the stump the form of a projecting cone. It is to the base of this cone,—that is to say, at the level of the retracted muscles and integuments, that the cutting instrument is applied, and the whole of the projecting part is removed. In thus withdrawing the flesh as it is divided, and cutting the projecting mass, " the bone may be denuded to the extent of six inches." " By this method the operation is performed with an astonishing rapidity: the operator preserves as large a cushion as he wishes for the bone, and he spares the patient the acute suffering occasioned by the successive dissection of tiguments and muscles." Whether this method be preferable or not, it is only common justice to the dead to state fairly the reasons which induced him to propose this mode of operating. We have read through the article " Amputation" in the *Leçons Orales* of M. Dupuytren; we have been unable to find any directions for raising up the thigh to the perpendicular; for the " ingenious reasons" quoted, or indeed any other.

The revival of the *High Operation for Stone*, Sir C. Bell tells us, " was on the alleged necessity arising from the difficulty of extracting large stones from below." The operation was invented by Franco, it is true, under such a necessity, which occurred in cutting a child of two years, from whom he was unable to extract the stone through the perineum; but this was not the reason for its revival: it was believed that the bladder was more accessible, and that no important organs intervened; and, certainly, in the hands of Souberbielle, the operation has had great success. Neither, we think, is our author correct in assigning the reasons which are used to obviate the objections to this operation. " The incision," he says, " is made first in the perineum, in the usual manner, then a staff is introduced from below, and the fundus of the bladder pushed up above the pubis: here it is cut upon, and the stone extracted." Frère Cosme, no doubt, made such a proposal, but it was not long followed; it was energetically opposed by Scarpa; and is, so far as we know, completely abandoned.

Our author's opinion on the subject of *Aneurism* is similar to that of Scarpa, Hodgson, and others: nevertheless, we believe that we are justified in maintaining that the time is come for some modification of the opinion of Scarpa. There are now on record many well-observed cases, in which there has existed a dilatation or pouch occupying *only a portion of the circumference of the tube*, where the point of communication with the vessel is *somewhat constricted in the form of a neck*, and where *a large coagulum filled this pouch*, whilst the most perfect continuity of the internal tunic was demonstrated. Had Scarpa been aware that such cases were occasionally observed, his definition of aneurism would have been different.

We were hardly prepared to expect that, in the perusal of these volumes, we should have discovered a remedy for *tic;* yet so it would seem: our author states that, since he has exhibited a compound of one drop of croton oil and one drachm of compound colocynth pill, divided into twelve pills, one of which, with ten grains of galbanum pill, are to be taken at bedtime, " every true case of tic which has been presented to him has been cured." Sir Charles, we presume, does not mean that the curability of the disease by this medicine is to be the test of true tic: if so, we fear that the definition will not be very currently received. What is *true tic*? Is it a nervous pain dependent on torpor or accumulation in the bowels? Usually, we believe, it is not so; yet we apprehend this is our author's true tic. We have ourselves tried this remedy exactly as prescribed by Sir C. B., in what was certainly *tic*, but apparently not *true tic*, since the results obtained by us by no means corresponded with those obtained by him.

The author's opinion upon the nervous system has most deservedly great weight; yet we cannot quite accord with the opinion stated in the following sentence:

"When a nerve is divided across, it cannot be perfectly restored; it unites, but a thousand chances are against its union—filament to filament. Sensibility is in part restored, but it is neither a perfect nor a natural sensibility which returns; a somewhat painful vibration is conveyed instead of the perfect sense of touch." (vol. ii. p. 107.)

It is very certain that union takes place upon the same principle and by the same means, in nervous as in other structures, and that the new tissue almost always, at the end of a period of time, varying with the tissue, acquires similar physical characters, and performs similar functions to the original structures. Arneman does not acknowledge that any regeneration takes place; and regards the substance which unites the nervous extremities as absolutely incapable of transmitting sensations; and this is equally denied by Breschet, by Richerand, and by Delpech. We apprehend they are in error. Fontana, after some time, examined the pneumogastric nerve of a rabbit, of which he had made a section, and found that the nervous filaments had no interruption. Michaelis examined nerves from which he had removed nine to twelve lines; and, by the aid of a microscope, could readily distinguish nervous filaments. Similar observations were made by Mayer; and he tested the truth by immersing the part in nitric acid, which produced no change. The observations of Cruickshank, Haighton, Prevost, and Swan are equally conclusive as to the recovery of structure and function. Tiedemann's researches into the recovery of function are equally satisfactory. Pring and Descot, Balfour, Peacock, Braid, Bailey, Houlton, and others, have given us conclusive illustrations of similar recovery of function in the human subject. Hunter, Fauchard, and Mouton, have left us instances of transplanted teeth acquiring sensibility. A large number of cases of tic might be cited, where a portion of nerve has been removed and the pain has ceased, but in a few weeks has again acquired all its former intensity. We do not, however, for one moment seek to maintain that the two portions unite filament to filament: in no tissue does this occur; but in all cases, we believe, the intervening or connecting structure acquires the character of those it connects, whether osseous, vascular, or nervous.

In the section on *Wounds of the Thorax*, it is said (vol. ii. p. 141,) "as to wounds of the heart and great vessels, I need not fill my pages with narratives; they are fatal wounds." Now this is not exactly the fact; and such an impression as is here conveyed should not be disseminated. The dictum of Hippocrates (*Aphor*. Sect. 6, No. 18,) is, that, without exception, wounds of the heart are necessarily mortal; it is also that of Celsus and of Paulus Egineta: yet a large number of cases are opposed to it. Wounds of the heart present a remarkable difference, dependent upon their situation, as well as upon the depth to which they have penetrated: if the cavities escape, the wound may excite only an inflammation, which may be subdued. Among many other examples furnished by comparative pathology is that related by Weber, where a musket-ball was found in the parietes of the heart of a stag; also one, contained in the Ed. Med. and Surg. Journal, (vol. v. 417,) in which a fat and vigorous doe was killed, in whose heart was found encysted a ball weighing 292 grains. In the human subject, among others, are the cases of Chabrol, Fourly, Richerand, and Latour: in one case, six years after the receipt of a gun-shot wound in the chest, the man died, and the ball was found in the right ventricle. We might mention many other cases, were it necessary.

In chap. viii., *Of Wounds in the Neck and Throat*, a curious anecdote is told, and is very properly held up to the student as affording a useful hint.

> "My predecessor in the Middlesex Hospital being under the hands of the barber, they began to talk of an attempt at suicide in the neighbourhood; on which the hospital surgeon called the wretched man a fool, and told the barber how he should have done it. The unfortunate barber retired into the back area, and cut his throat: there was no saving him!" (p. 149.)

An observation respecting the introduction of air into the blood-vessels would suggest some remarks; but we intend noticing this subject in another place.

We pass over chap. ix., *On Diseases of the Eye and the Operations performed on the Eye*, for the same reason.

Sir C. Bell seems to think that no satisfactory explanation can be given of the mode of formation of *loose cartilages in the knee-joint.* "It is sufficient to our present purpose, (he says,) to observe that they can be traced to a remote inflammation in the joint." Now, we think that the mode of their formation is satisfactorily made out, and that very commonly they cannot be traced to a remote inflammation. An opinion formerly entertained was, that they were masses of exfoliated articular cartilages. Loose cartilages, so formed, unquestionably have existed, but very unfrequently. Hunter believed them to be produced from extravasated blood which became organized and transformed. Russell thought they resulted from the condensation of a certain portion of synovia. There cannot, however, be a question that ordinarily they are differently formed: they are developed in the cellular tissue external to the synovial tunic; in the progress of development, they push before them that tunic and become pediculated; their pedicle becomes thinned, ruptured, and the body is at length free in the articulation. In Cruveilheir's *Anat. Path.* (Livraison 9, pl. 6,) there are many of these bodies represented in successive stages of development.

The last chapter of the work is on *Syphilis;* and opens with the following striking paragraph.

> "Is there any experienced senior of the profession, who, having a son of eighteen or twenty, and that son having a chancre, that would treat him without mercury? No! there is not such an unnatural person. This is our text, for to this the practical question should be brought." (p. 228.)

We have lately discussed the subject of the different modes of treating syphilis so fully (See Br. and For. Med. Rev., Vol. VI. Art. I.), that it is unnecessary to enter upon it here; but we cannot help remarking that here, as in many other parts of his work, Sir Charles Bell is disposed to be too dogmatical or too exclusive. In speaking of the cure of chancre, he says, "It is a matter proved that the primary sore will, in certain circumstances, and by treatment without mercury, heal; but it is as certain that, so treated, a sore-throat will follow." (p. 242.) Now, we think that the statements in the article referred to prove that this opinion, and this assertion, are too lightly advanced. There cannot, we apprehend, be a question in the present day that all the primary, and many secondary, symptoms of syphilis may be cured, and that, too, in a manner as complete as durable, without having recourse to any specific means, mercurial or other. It also has been proved that the secondary or constitutional symptoms occur, little if at all, more frequently than after the treatment by mercury; and it is doubtful whether these symptoms are ever so aggravated and obstinate. Indeed, the mode of treatment recommended by many of the most enlightened surgeons of the present day, is, first, to employ an antiphlogistic treatment for a reasonable time, and, if the sore remains obstinate, then to use mercury moderately.

But it is time to bring this article to a conclusion; and, in doing so, we must express our sincere regret that our task has been so much that of the critic. Our feelings towards the illustrious author would have led us to pass over defects and enumerate only excellencies; but the sterner duties of our office did not allow of this. Many things which we have criticised in him, we would have left unnoticed in the work of an inferior or unknown author. Sir Charles Bell's authority is potent for good or for evil; and it is our duty to use the humble means in our power to enhance the former and mitigate the latter. Without reference to former writings which have created the authority of which we speak, it is sufficiently manifest, from the work before us, that he who could write several of its chapters could have written all well, had he seen fit to use the exertion necessary for the purpose. It is a matter, not only of deep regret, but a probable source of serious evil, that this exertion has not been made.

---

